# Mass drug administration for endemic scabies: a systematic review

**DOI:** 10.1186/s40794-021-00143-5

**Published:** 2021-07-01

**Authors:** Giulia Rinaldi, Kholoud Porter

**Affiliations:** Institute for Global Health, University College London, London, UK

**Keywords:** Scabies, Mass drug administration, Endemic

## Abstract

**Background:**

Scabies is an extremely fastidious infestation caused by the Sarcoptes scabiei mite. It causes a persistent itch that can disrupt a person’s mental health, sleep, and overall quality of life. In endemic areas, treatment by targeting symptomatic individuals and their contacts is often unsuccessful due to an asymptomatic period and high rates of re-infection. To overcome this, Mass Drug Administration (MDA) is often used to treat the whole community, irrespective of whether individuals presently have scabies. This review summarises the evidence for the effectiveness of MDA in treating scabies.

**Methods:**

An exhaustive literature review was conducted on MEDLINE, EMBASE, Web of Science and Scopus. All peer-reviewed articles published in English January 1990 to March 2020 were eligible and only if the studies were primary and interventional. Furthermore, the intervention had to be a pharmacological MDA method involving human subjects.

**Results:**

TWELVE articles that qualified for inclusion were identified. MDA for scabies significantly reduced its prevalence in communities at follow up. Some of the drivers of success were communities with low levels of migration, an uptake of MDA of > 85%, the use of oral Ivermectin therapy, the treatment of children and pregnant women within the treated population, and repeated treatment for participants diagnosed with scabies at baseline.

**Conclusions:**

The average absolute reduction in prevalence of scabies was 22.0% and the relative reduction average was 73.4%. These results suggest MDA is effective in treating scabies in the endemic community. Further evidence is needed surrounding MDA use in urban areas with increased levels of migration. Importantly, MDA should not substitute the tackling of socioeconomic factors which contribute to endemic disease such as good sanitation and hygiene.

**Supplementary Information:**

The online version contains supplementary material available at 10.1186/s40794-021-00143-5.

## Introduction

### Health implications

Scabies is a parasitic skin infestation caused by *Sarcoptes scabiei* which has infected humans for thousands of years and remains a globally pressing issue in both humans and livestock [1]. *Sarcoptes scabies* belongs to the Sarcoptoidea family of mites which secrete a unique saliva that allows them to penetrate the stratum corneum and move their claws in a quick swimming motion to burrow into the epidermis [2]. They are difficult to eradicate; mites can survive on fabrics for up to 3 weeks and require washing temperatures of over 50 degrees Celsius to ensure killing [3]. Additionally, symptoms often lag 1 month behind initial contraction of the mite generating asymptomatic spreaders. Scabies is an extremely fastidious infection and the constant itch can affect a person’s mood, sleep, concentration, work and overall quality of life [4]. Most individuals will present with the typical rash including erythematous papules, nodules and serpiginous “burrows” [5]. The commonest areas for these skin changes to appear is between the finger webs, the ankles, the genital areas and the elbows [1]. However, some individuals can present with clear itchy skin or medical complications of scabies.

The medical complications of scabies include skin infections and glomerulonephritis [6]. One of the commonest complications is impetigo; a bacterial skin infection caused by staphylococcus or streptococcus bacteria. The close relationship between the prevalence of scabies and impetigo is well known [7]. In areas where there is poor access to antibiotics, skin infections can easily spread along the epidermis and cause cellulitis, sepsis or glomerulonephritis. Rarely, scabies can manifest as crusted “Norwegian” scabies which is a rare, severe hyperkeratotic reaction to a hyper-infestation of scabies associated with frailty and immunosuppression [5]. The mental health implications of scabies include low mood associated with constant itch and lack of sleep, and, the consequences of stigmatisation and isolation from their community [4]. Investigators of a study in Brazil reported that around 77% of adults with scabies feel shame and over 65% feel that it had reduced their quality of life [8].

### Epidemiology

It is estimated that there are around 300 million people infested with scabies at any point in time [9]. In high income counties, for example the United Kingdom, the overall prevalence of scabies is deemed to be low with figures estimating between 2.2–2.8 per 1000 population.

**Fig. 1:**
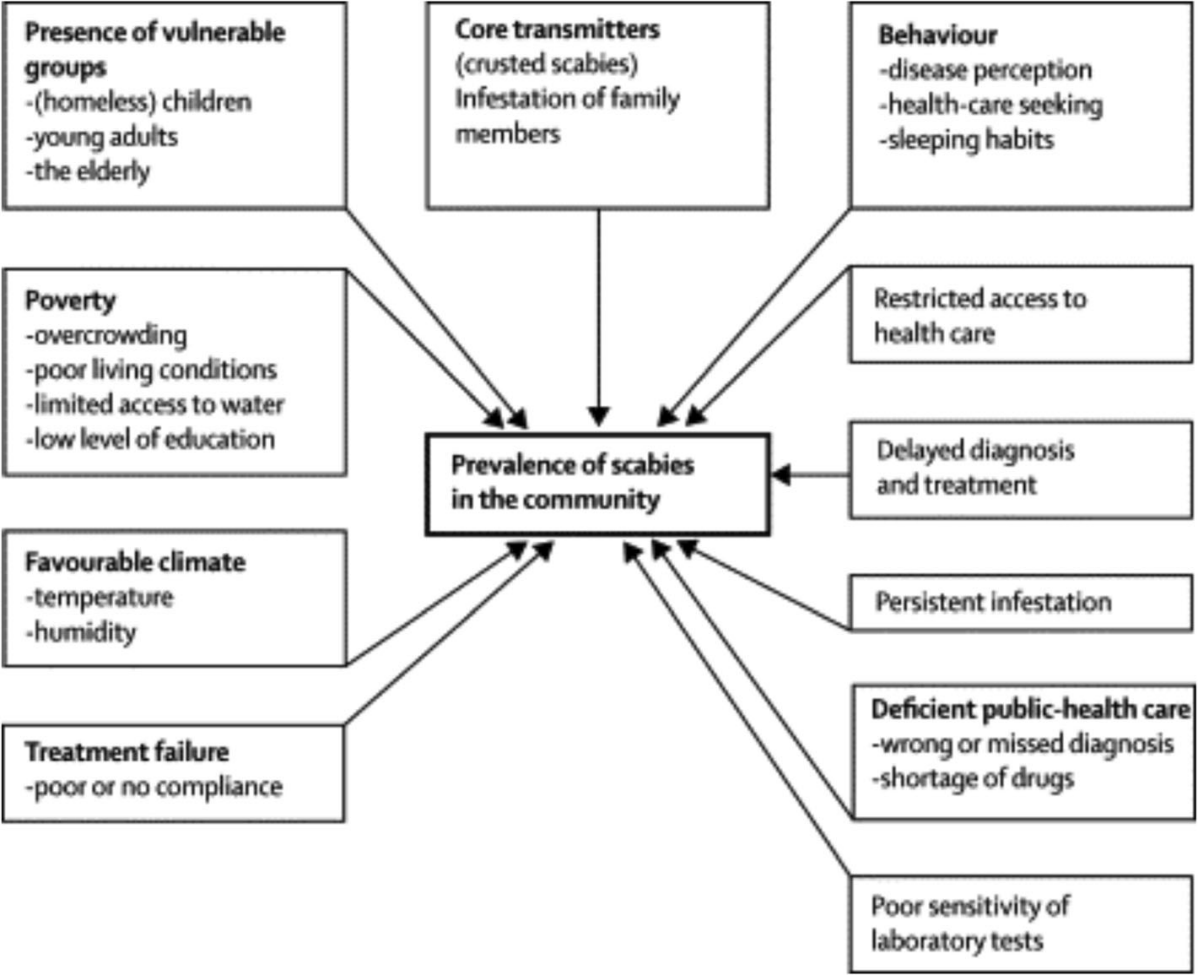
Factors contributing to a high prevalence of scabies in resource-poor communities [10]

In low- and middle-income countries, scabies remains one of the most common skin diseases and causes a high burden of morbidity. The socioeconomic factors most often associated with endemic scabies are represented in Fig. 1. The prevalence of scabies is highly variable based upon setting, so there are wide variations in estimates of prevalence. The prevalence of scabies amongst different communities has been estimated from 0.4% in Turkish urban pre-school children to 39.1% in specific districts in Timor-Leste, and as high as 83% in rural households in Kerala, India [9, 11]. Landwehr et al. compared scabies prevalence rates amongst rural and urban children in Mali, Malawi and Cambodia [12]. They consistently found that children from higher socioeconomic backgrounds had lower rates of scabies. Importantly children from households with overcrowded sleeping habits and farming families had a higher prevalence of infestation. In fact, lower socioeconomic status is likely a confounder due to its association with overcrowding, poor sanitation, and unclean water supplies [13].

The 2016 Global Burden of Disease (GBD) study estimated that scabies accounted for 0.21% of all Disability adjusted life years (DALYs) [10]. In 2017 scabies accounted for 59.27 DALYs per 100,000 globally [14] and was responsible for more global age standardized DALYs than atrial fibrillation and acute lymphocytic leukaemia [10]. This morbidity directly impacts the work and income that individuals can obtain often creating increased socio-economic deprivation amongst those who are already disadvantaged. Scabies infestations and outbreaks can also create large costs for healthcare systems. Treating an outbreak of scabies can be very costly, in one Canadian care home with 387 residents the cost was estimated at $200,000 [15]. In resource poor settings, where outbreaks can involve whole communities, the costs for treatment and isolation can be unaffordable for the country’s healthcare system.

### Treatment of scabies

The treatment of scabies is challenging and requires rigorous adherence. Most treatment for scabies is prescribed on an individual basis to the patient with symptoms. This will often consist of a topical regimen, usually permethrin 5% cream, which has to be applied all over the body on 2 days 1 week apart [16]. In resistant cases, especially crusted scabies, oral Ivermectin is usually prescribed as two single doses 7 days apart [6]. However, in overcrowded and resource deprived areas the diagnosis of scabies is often delayed, and the patient’s contacts may already be infected. In endemic areas, individual treatment is often futile and wastes valuable resources due to high rates of re-infection.

Mass drug administration (MDA) is the administration of treatment to a whole community regardless of whether individuals currently display signs of infection [17]. This method is gaining popularity to target scabies outbreaks within communities that have high prevalence rates. Over the last decade, the evidence surrounding MDA for endemic diseases has grown quickly with increasing communities trialling this method [17–19]. The regimens for MDA for scabies are often comprised of either treatment with topical permethrin 5% or oral ivermectin [20]. The World Health Organisation (WHO) currently recommends mass drug administration for scabies in populations where prevalence is > 10% [21]. An exhaustive systematic review on available published evidence is lacking, so the most recent WHO recommendations published in 2020 are based on results from a handful of studies and highlight the need for a review of available evidence [21]. Moreover, nature of MDA programs signifies that these interventions are of large scale, therefore, it is crucial to understand and analyse the current evidence to guide future establishment of MDA programs for scabies.

As evidence surrounding MDA for scabies is growing there is an increased need to summarise and analyse the data available on this topic. Specifically, to explore program characteristics leading to the greatest reduction in scabies prevalence. To our best knowledge this is the first review to summarize the evidence surrounding the use of MDA and to identify their driving factors of success.

## Methods

### Search strategy

A comprehensive literature review of four databases was performed. These included MEDLINE (Pubmed), EMBASE (ovid), Web of Science (core collection) and Scopus. A search of the grey literature was conducted on google scholar, the World Health Organisation (WHO) website, and through references of the included papers. The literature search was performed on the 24th March 2020. The initial search strategy was created on MEDLINE and then adapted to the formats of the included databases. The search consisted of keywords divided into three main concepts; “scabies”, “effectiveness” and “mass drug administration”. Boolean operators “OR” and “AND” were used to combine the different keywords into the search strategy. The full search strategy, in the MEDLINE format, is included in Additional file 1: S1 Appendix.

### Eligibility criteria

Included studies had to deliver MDA; defined as the treatment of a whole community, both adults and children, regardless of whether individuals had clinical evidence of scabies. Studies had to be interventional, evaluating the prevalence of scabies before and after MDA. Publication had to be between January 1990 to March 2020 to increase relevance of results. All settings of studies identified, including low, middle- and high-income countries, were included in this review. Studies that executed non-drug MDAs, such as educational or environmental methods of scabies control, were excluded. Additionally, studies had to involve human subjects; biological and theoretical models were excluded. Only the first publication of each study was included to avoid bias. Findings from trials’ longer-term follow up results are, however, included in the discussion section. Other exclusion criteria included articles that were not published in the English language or not published in a peer reviewed journal. Unpublished documents, conference abstracts, and dissertations were also excluded.

### Screening

Papers from the database search were exported into Covidence an online systematic review program to facilitate the study screening and selection process [22]. The identified papers were first searched for duplicates then the remaining papers had their titles screened to allow for quick elimination of studies that did not fit the inclusion criteria. Subsequently, we screened the full text of remaining articles for appropriateness against the inclusion and exclusion criteria.

### Data extraction

The extracted data included authors, country and the study design. The setting included whether it was an urban or rural, the population size and the income level described (upper, middle or lower) as per the 2020 World Bank thresholds [23]. The medication regimen used was noted including the pharmaceutical agent, the dose, and whether alternatives were given to those with contraindications to the main treatment.

The baseline prevalence of scabies, how it was diagnosed and what percentage of the population underwent examination was recorded. The percentage of the total population which underwent examination at follow-up was also recorded. The time interval from MDA to follow up was noted. If the studies used an interrupted time series design with multiple follow up periods, the outcomes at 12 months were used in the analysis to allow for greater homogeneity amongst the results. The prevalence of scabies prevalence before and after MDA was compared statistically using an unpaired t-test. In addition, secondary outcomes analysed in the studies were noted regarding any spill over effects of the MDA programs. If studies evaluated adverse effects of the treatment, then these were also recorded. Lastly, factors that may have contributed to the success of interventions were noted and any mentioned limitations of the interventions also recorded.

### Quality of evidence

The quality and risk of bias of the included studies was assessed using the Risk of Bias in Non-Randomized Studies – of Interventions (ROBINS-I) assessment tool [24]. As a majority of our included studies are single arm, we have edited the ROBINS-I tool to not assess for participant selection and allocation bias. Our modified ROBINS-I tool is presented in Additional file 1: Appendix S2. This version assesses risk of bias in 6 domains including confounding, intervention classification, deviation from intended intervention, missing data, outcome measurement and selection of the reported result. The risk of bias was assessed as having a low, moderate, serious or critical risk in each domain. The overall risk of bias was recorded as the highest level of bias detected in that category amongst all included studies.

## Results

### Study search

**Fig. 2:**
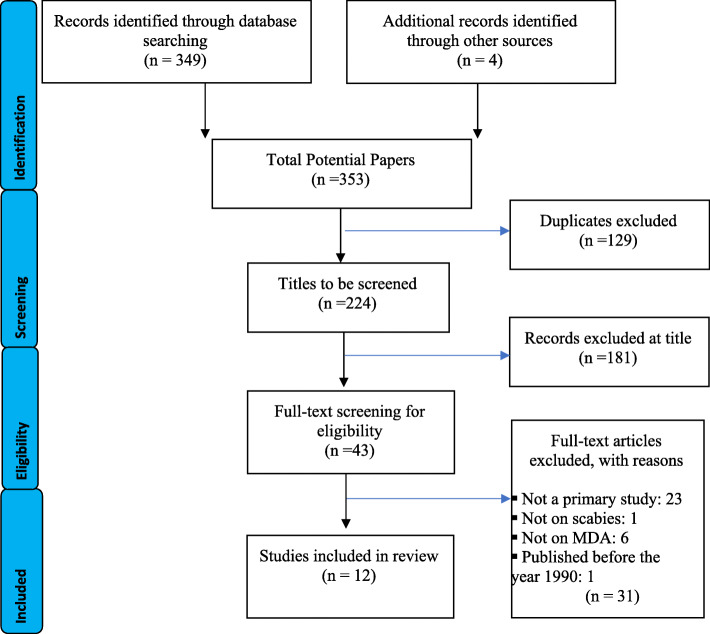
PRISMA Study search process

The database search identified 353 publications. After the exclusion of duplicates and title screening 43 articles remained. After full text screening, 1012 articles were identified for inclusion in the final analysis. A total of 31 articles did not fit the review criteria as 23 were not primary studies, one was not about scabies, six did not use MDA and one was published before 1990. The study published prior to 1990 administered lindane lotion which is no longer recommended for the treatment of scabies. The study selection is represented using the preferred reporting items for a systematic review and meta-analysis protocol (PRISMA) in Fig. 2.

### Included studies

Amongst the 12 included papers three were set in the Solomon Islands [25–27], two in Fiji [28, 29], two in Tanzania [30, 31], three in Australia [13, 32, 33] and one in both Papa New guinea [34] and Panama [35]. All 10 studies were conducted in rural settings amongst small village communities. The population sizes ranged from 120 to 5000 people. The total number of participants from all included studies was 20,000 (to the nearest 100). All the studies were prospective and interventional. The time from MDA administration to follow-up examination ranged from 24 days to 4 years, with seven studies providing follow up results for the time period between 1 and 2 years after MDA. The general features of the included papers are represented in Table 1.

**Table 1:** General features of included papers

Author	Year	Setting	Population Size (nearest 100)	Alternative Treatment for Individuals with Contraindications	Further Treatment for Individuals with Clinical Scabies	Drug Delivered	Baseline Scabies Prevalence	Months after MDA for follow up Examination	Scabies Prevalence after MDA
**Andrews** **[**13**]**	2009	Rural Australia	5000	All Eligible	No	Topical Permethrin	16.10%	12 months	13.4%
**Bockarie** **[**34**]**	2000	Rural Papa New guinea	Arm 1–30 Arm 2–60	No	No	Arm 1 -Oral Ivermectin Arm 2- No treatment	Arm 1–87% Arm 2–52%	5 months	Arm 1–26% Arm 2–60%
**Carapetis** **[**33**]**	1997	Rural Australia	200	No	No	Topical Permethrin	25%	1 month	6%
**Haar** **[**28**]**	2014	Rural Fiji	Cluster 1: 600 Cluster 2: 700	Yes	No	Cluster 1- Oral Ivermectin Cluster 2- Topical Benzyl Benzoate	Cluster 1–23.7%, Cluster 2–37.9%	Cluster 1: 24 days, Cluster 2: 28 days	Cluster 1–20.0% Cluster 2–9.5%
**Kearns** **[**32**]**	2015	Rural Australia	1200	Yes	Yes	Oral Ivermectin	4%	18 months	2%
**Lawrence** **[**27**]**	2005	Rural Solomon Islands	1600	Yes	Yes	Oral Ivermectin	20%	24 months	1%
**Leppard** **[**30**]**	2000	Rural Tanzania	1200	All Eligible	Yes	Oral Ivermectin	71%	12 weeks	0%
**Marks** **[**25**]**	2019	Rural Solomon Islands	Arm 1–700 Arm 2–700	Yes	Yes	Arm 1- Oral Ivermectin Arm 2- Oral Ivermectin plus Azithromycin	Arm 1–11.8% Arm 2–9.2%	12 months	Arm 1–1.0% Arm 2–0.7%
**Martin** **[**31**]**	2018	Rural Tanzania	4000	Yes	No	Oral Ivermectin	4.4%	12 months	0.84%
**Romani 2015** **[**29**]**	2015	Rural Fiji	Arm 1–800 Arm 2–500 Arm 3–700	Yes	Yes	Arm 1- Standard care (affected people & their household contacts) Arm 2- Topical Permethrin Arm 3- Oral Ivermectin	Arm 1–36.6% Arm 2–41.8% Arm 3–32.1%	12 months	Arm 1–18.8% Arm 2–15.8% Arm 3–1.9%
**Romani 2019** **[**26**]**	2019	Rural Solomon Islands	1400	Yes	No	Oral Ivermectin and Oral Azithromycin	18.70%	12 months	2.3%
**Taplin** **[**35**]**	1991	Rural Panama	800	No	No	Topical Permethrin	33%	1 month	2.5%

Two of the study designs were randomised, including two different MDA regimens, assigned randomly to different rural villages [25, 29]. Additionally there was one further study that was controlled, albeit not randomised, which included one interventional MDA village and one control village with no intervention [34]. The remaining 9 studies were either single-arm or parallel-arm interventional studies without a control arm.

### Characteristics of MDA regimens

**Fig. 3:**
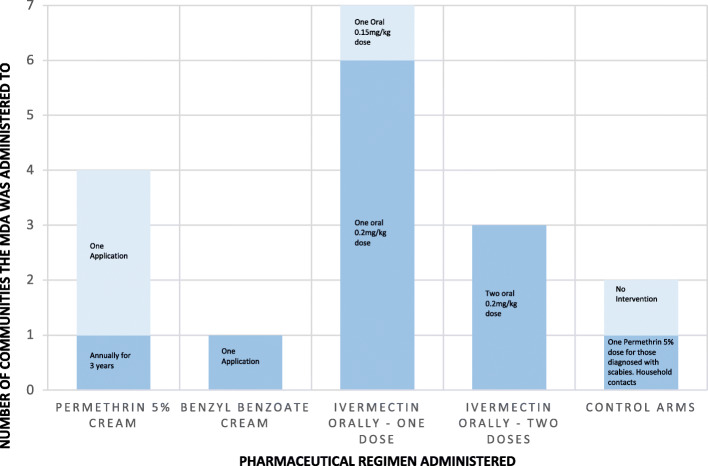
Drug regimens included in Mass Drug Administration of scabies

The included 12 studies were performed in 17 communities receiving treatment regimens for scabies as represented in Fig. 3. The most common MDA regimen was a one-off dose of oral Ivermectin, with seven communities receiving this. Of these, six administered a dose of 0.2 mg/kg and one administered a dose of 0.15 mg/kg. The second most common MDA regimen was topical permethrin 5% cream with four communities receiving this. Three communities administered this once, whilst one community administered this annually for 3 years [13, 29, 33, 35]. Next most common was two oral doses of Ivermectin at 0.2 mg/kg with three communities receiving this. Of these, two communities received the doses 7 days apart, whilst the third community received them 12 months apart.

One study administered a one-off dose of benzyl benzoate cream to their community. There was one, non-MDA, control arm in Romani et al’s study which involved those diagnosed with scabies and their contacts receiving a one-off dose of Permethrin 5% cream [29]. Lastly, another study had a control arm village which received no intervention for scabies [34].

Participants in four communities shown in Fig. 3 that used topical permethrin 5% or benzyl benzoate, including small children and pregnant women, were safe to receive this treatment. Similarly, in one Ivermectin based study set within a prison, all the inmates were male and eligible to receive the oral Ivermectin treatment [30]. However, in the other nine communities’ alternative treatment was provided to individuals for whom Ivermectin was contraindicated. In eight of these studies, permethrin 5% cream was given to pregnant or breastfeeding women or children who weighed under 15 kg. In two communities, those ineligible for treatment, including young children and/or pregnant/breastfeeding women were given no treatment [34].

Five of the 12 included studies offered additional treatment adjunctive to the MDA regimen for those that were diagnosed as having scabies at baseline population screening. In three of these, a second dose of the initial MDA treatment was administered between 7 and 14 days after the first [25, 29, 31]. In two of these studies, the participants diagnosed with scabies were followed up at 8 weeks and 3 months respectively for a second treatment. In the study with 8-week follow up, if patients persistently had clinical scabies after the single ivermectin dose failed, they were given 1% lindane lotion and environmental cleaning of their cell and clothes [30]. In the 3 month follow up study, those with persistent scabies after two oral ivermectin doses were given a third ivermectin dose along with their household contacts [27].

#### Effectiveness of MDA

All 15 of the communities that received MDA for scabies showed a reduction in the prevalence of scabies at follow up. The average absolute reduction in prevalence of scabies was 22.0% (*p* = 0.0003) and the relative reduction average was 73.4%. The control community in Bockarie et al’s study was the only one that did not experience a reduction of scabies prevalence at follow up [34]. The baseline and follow up prevalence of scabies are presented for each community in Table 2.

**Table 2:** Prevalence of Scabies - Before and After MDA

Community Number	MDA Regimen	Author	Prevalence of Scabies Before MDA	Prevalence of Scabies After MDA	Community that Received MDA (%)	Prevalence Absolute reduction (%)	Prevalence Relative reduction (%)
1	**Control**	**Bockarie et al.**	52.0%	60.0%	n/a	−8.0%	−15.4%
2	**Standard Care** **(control)**	**Romani et al. 2015**	36.6%	18.8%	n/a	17.8%	48.6%
3	**BB Cream**	**Haar et al**	23.7%	9.5%	76.0%	14.2%	59.9%
4	**Permethrin**	**Romani et al. 2015**	41.8%	15.8%	85.0%	26.0%	62.2%
5	**Permethrin**	**Andrews et al.**	16.1%	12.7%	Not Reported	3.4%	21.1%
6	**Permethrin**	**Carapentis et al.**	25.0%	6.0%	Not Reported	19.0%	76.0%
7	**Permethrin**	**Taplin et al.**	33.0%	3.6%	99.7%	29.4%	89.1%
8	**IVM Two oral doses**	**Kearns et al.**	4.0%	2.0%	96.0%	2.0%	50.0%
9	**IVM Two oral doses**	**Romani et al. 2019**	18.7%	2.3%	99.0%	16.4%	87.7%
10	**IVM Two oral doses**	**Lawrence et al.**	20.0%	1.0%	95.0%	19.0%	95.0%
11	**IVM Single oral dose**	**Haar et al.**	37.9%	20.0%	49.0%	17.9%	31.1%
12	**IVM Single oral dose**	**Bockarie et al.**	85.0%	26.0%	87.0%	59.0%	69.4%
13	**IVM Single oral dose**	**Martin et al.**	4.4%	0.8%	85.0%	3.6%	80.9%
14	**IVM Single oral dose**	**Marks et al.**	11.8%	1.0%	91.0%	10.8%	91.5%
15	**IVM Single oral dose**	**Marks et al.**	9.2%	0.7%	91.0%	8.5%	92.4%
16	**IVM Single oral dose**	**Romani et al. 2015**	32.1%	1.9%	85.0%	30.2%	94.1%
17	**IVM Single oral dose**	**Leppard & Naburi**	71.0%	0.1%	100.0%	70.9%	99.9%
					**Average** **(Excluding Community 1&2)**	**22.0%**	**73.4%**
					**T-Test (*p*-value)**	**0.0003**	

Two studies, represented as community number 5 and 6 in Table 2, did not report the percentage of the population that successfully received MDA. Moreover, the community represented as number 1, is the control arm in Bockarie et al’s (2000) study, which was the only community that received no intervention; therefore, this result is represented as 0%. Community number 2, is a non-MDA standard care arm from Romani et al.’s (2015b) study.

#### Oral MDA regimens

Ten communities received oral MDA regimens. The seven communities that received a single oral dose of Ivermectin all experienced a reduction in their scabies prevalence at follow up. These are represented as community 8–17 in Table 2 and all experienced a large reduction in prevalence of scabies. Similarly, amongst the communities which received two oral doses of Ivermectin the baseline scabies prevalence dropped; these are represented as communities 8 & 9 in Table 2. The follow up intervals varied from 24 days to 24 months with the majority (8 out of 10 communities) having a follow up interval between 12 and 24 months.

#### Topical MDA regimens

Five communities received a topical MDA regimen which are represented as community 3–7 in Table 2 [13, 28, 29]. The specific regimens were a single dose of Permethrin 5% cream, three doses of Permethrin 5% cream 12 months apart and a single dose of Benzyl Benzoate cream. The time interval between the scabies prevalence before the first MDA and the last follow up ranged from 1 month to 3 years.

#### Non-MDA controls

Two communities included in this review did not receive MDA. The first one was the control village, in Bockarie et al’s (2000) study, that received no intervention or treatment represented as community 1 in Table 2. At baseline the prevalence of scabies in this community was 52% and, at follow up, after 5 months it was estimated to be 60% [34]. The second community from Romani et al’s 2015 study, represented as 2 in Table 2, received a control intervention involving treating those diagnosed as having scabies and their contacts with a single dose of permethrin 5% cream. This community had a baseline scabies prevalence of 36.6% and at 12 months after follow-up had a scabies prevalence of 18.8% [29].

### Secondary outcomes

Seven reported other outcomes of MDA. The most common outcome reported was the effect of MDA on the prevalence of bacterial skin infection. Seven of the included studies reported the baseline of skin infections before and after intervention [13, 25–27, 29, 33, 35]. They reported an average absolute decrease of 16.7% and relative decrease of 68.0% in the prevalence of infected skin lesions. One study also recorded the change in number of children found to have haematuria before and after MDA for scabies and found that there was a significant decrease in number of children with haematuria after receiving scabies treatment [27].

### Adverse effects

Ten studies (83%) recorded adverse effects from MDA treatments. None of the participants in these ten studies experienced any serious adverse events, defined as, any effect that would require medical attention or did not resolve within 7 days. Due to this, four of these studies stated that no significant adverse effects were noted, but did not explore mild adverse effects. The remaining six studies recorded the characteristics of the mild adverse events. These were experienced at a prevalence ranging from 0.0–15.6% [26, 28–30]. In one single-dose oral ivermectin group the adverse events were reported as high as 15.6% [29]. The most common adverse event was itching, followed by headache. In the single dose permethrin group, the highest adverse event rate reported was 6.8% with the commonest symptoms also being itching and headache. In a topical benzyl benzoate three-times daily group, 9.5% of participants reported mild adverse events were itching, stinging and burning [28]. One study reported that none of its participants complained of mild side effects [30].

### Quality of evidence

The results from our modified ROBINS-I tool are summarised in Table 3. The first domain Bias in Confounding was met poorly by the majority (83%) of the included studies. The main reasoning for this is that these were non-randomised non-controlled trials which allow for a higher chance of confounding compared to randomised controlled trials (RCTs). It is important to highlight that the majority of evidence in this review is from single-arm interventional trials and there is a lack of RCTs in this field.

**Table 3:**
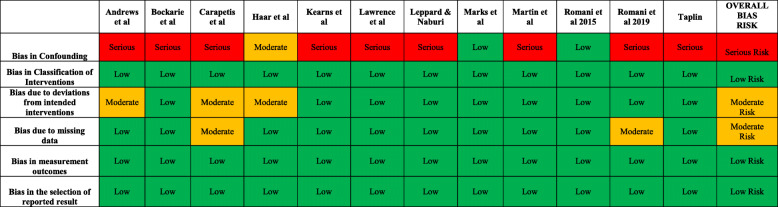
Quality Assessment of Included Evidence

The classification of interventions, selection of reported results and measurement outcomes had low risk of bias with all included studies having low risks in these categories. This is due to the clear definition of the MDA regimens and the clear outcomes stated in the included studies. There were two categories with a moderate risk of bias; deviation from intended interventions and missing data. The bias in deviation from intended interventions is because two studies did not report what percentage of the population successfully received MDA [13, 33] and one had less than 80% of their population successfully receive it [28]. There were also two studies that did not report what percentage of the population were clinically examined at follow up [26, 33], therefore, these resulted a moderate overall risk in the missing data section.

Moreover, the included studies were evaluated for sources of funding and conflicts of interest statements. All of the articles included a statement to acknowledge sources of funding and 6 (50%) of articles included a conflicts of interest statement.

## Discussion

### Interpretations of findings

In this review, the first of MDA as treatment for scabies, our findings highlight that it is successful at significantly reducing the prevalence of scabies within communities supported by high quality evidence.

Every community in this review which received MDA experienced a decline in the prevalence of scabies at follow up. The average absolute reduction in prevalence of scabies was 22.9% and the relative reduction average was 73.4%. Although, the included studies were of high quality, the majority were single-arm interventional trials. Nevertheless, these findings strongly suggest that MDA regimens for scabies are successful and supports previous reviews of other conditions showing that pharmaceutical MDA regimens can successfully control endemic disease [36, 37]. In fact, one MDA regimen targeting onchocerciasis in Columbia has shown complete eradication of this disease [38].

### Drivers of effectiveness

The heterogeneity amongst the included MDA regimens originally created some difficulties with comparability, however, it also allowed for the identifications of drivers of success amongst a group of altogether successful interventions. There are many questions that remain to be answered regarding which specific characteristics of MDA programs enhance success. These include crucial questions including the thresholds of baseline prevalence of scabies, who should be treated, with what drug regimen, with how many cycles of MDA and whether intensive treatment is required for those that are diagnosed with active scabies.

The five communities, included in this review, that had the greatest relative reduction in scabies prevalence shared certain characteristics. These communities experienced a relative reduction in scabies prevalence ranging from 91.5 to 99.9% [25, 27, 29, 30]. Their populations were all rural with low levels of migration and the population sizes ranged from 700 to 1600. Importantly, all five of these communities experienced very good coverage of MDA, with 85 to 100% confirmed to have received the medication. In fact, the WHO recommends a minimum 65% coverage for MDA, but ideally programs should aim to target 80% of their population including children [39]. Programs with poor uptake or who do not monitor uptake have repeatedly been shown to experience lower levels of disease eradication [13, 28, 40]. The underlying question is how to logistically deliver the MDA to ensure the greatest uptake. One systematic review evaluating methods in schistosomiasis MDA concluded that community-based methods (e.g. household-to-household or from a central location) combined with school-based distribution were one method to maximise treatment uptake [41].

The type of pharmaceutical regimen also seems important in determining the success of the MDA program. All five of these communities used oral Ivermectin which may suggest that oral Ivermectin produces better results than topical treatments for scabies. Most evidence shows that Ivermectin and permethrin have equal efficacy in the treatment of scabies [42]. However, Ivermectin is much simpler to take correctly and can easily be directly observed within an MDA program. Therefore, this it appears that Ivermectin is more successful in MDA programs may be due to increased effectiveness instead of efficacy. In fact, one of the included studies that was less successful used permethrin 5% cream and did not directly observe the application of this amongst its participants, which may have contributed to its poorer outcomes [13].

These results highlight an important factor regarding the pharmaceutical regimen; that another driver of success of MDA interventions is to ensure that young children and pregnant women, who often have high rates of infestation, are also given an appropriate treatment. Out of the five identified most successful MDA communities one did not include any women or children because it was based in a prison [30]. This study was able to administer Ivermectin to every single participant and at follow up there were no cases of scabies. This strengthens the argument that Ivermectin is extremely successful in MDA programs targeting scabies, especially, if every participant receives a form of treatment. The other four studies treated all children under 15 kg and pregnant women with permethrin 5% as they were not eligible for Ivermectin. This highlights the importance of treating all members of a population during MDA, which may require alternate treatment options patients with contraindications to ivermectin such as children and pregnant women. Moreover, new evidence is being published demonstrating that Ivermectin is also safe amongst children weighing under 15 kg [43]. This will allow for a greater proportion of the population to receive Ivermectin, and perhaps, improve outcomes further.

Nevertheless, it is important to consider patient reported outcomes, such as side effect profile. As stated in the results section, ivermectin poses a greater risk of mild side effects such as itching and headache to occur. However, none of the participants included in this review experienced any serious side effects that needed medical attention. The high tolerability of these regimens suggests tolerability on a population basis is less important when choosing MDA regimen.

Out of these five MDA programs with the most successful outcomes, four of them only administered a single dose of Ivermectin. It would seem intuitive to believe that the three communities in our review that received double dosing of Ivermectin would have the best outcomes, however, this did not appear to be the case. On further analysis, the five very successful MDA communities had specific treatment pathways for those that were initially diagnosed with scabies at screening. This was only also the case in one out of the 12 other included communities. These communities all followed up those diagnosed with scabies a few weeks after the initial MDA administration for the administration of a second dose. Therefore, treating those diagnosed with scabies at baseline with a further dose after MDA might be a key to the success of MDA programs.

These findings suggest that the drivers of success for MDA are small communities with little migration, an uptake of > 85%, an Ivermectin regimen, the treatment of all population members (including children and pregnant women) and repeated treatment for participants with scabies at baseline.

### Spill over effects

The reduction of bacterial skin infections was equally demonstrated in both MDA regimens with oral Ivermectin and with topical permethrin. In one study, the arm that used both Ivermectin plus azithromycin versus Ivermectin alone, showed no difference in magnitude of the reduction of skin infections [25]. This could imply that successful pharmaceutical MDA programs provide a beneficial spill-over effect against skin infections without the need for adjunctive antibiotic therapy. An Ivermectin MDA program in the Solomon Islands demonstrated that Ivermectin significantly reduced the prevalence of haematuria amongst children in the treated community [27]. Whilst two studies demonstrated that after Ivermectin or Permetrhin 5% MDA for scabies the prevalence of head lice reduced significantly by 70.6% in one study and by 100% in another study [35, 44]. Lastly, Ivermectin treatment also showed an increased death rate of mosquitos signalling the possibility of an increased mosquitocidal effect of ivermectin [45]. Similarly, in Australia, Ivermectin MDA for scabies significantly reduced the prevalence of strongyloidiasis in the community [46].

In fact, African countries have been successfully delivering MDA programs over the last decade to target Onchocerciasis, lymphatic filariasis, schistosomiasis and soil transmitted helminthiasis [47–50]. There is growing evidence that MDA programs can successfully target multiple neglected topical diseases in communities where these are endemic [51, 52]. For example, Ivermectin is indicated for lymphatic filariasis, onchocerciasis, strongyloidiasis and scabies [53]. Furthermore, if Ivermectin is co-administered with another drug like Albendazole then the MDA program can also target Hookworm, Ascariasis and Trichuriasis.

A study in Zanzibar, showed that an Ivermectin and albendazole MDA targeting lymphatic filariasis, onchocerciasis and helminths also had a possible positive spill-over effect on the reduction of scabies infections due to a reduction in prescriptions for scabies treatment [54]. In multiple regions annual MDA with Ivermectin, albendazole and praziquantel has proven safe and successful in reducing the prevalence of lymphatic filariasis, onchocerciasis and schistosomiasis [55, 56]. Combined MDA programs would also create unprecedented logistical opportunities for current programs to link with new scabies MDA programs. Moreover, delivering triple drug therapy in one single sitting was shown to reduce costs by an average of 41% compared to delivering it during three MDA programs a few weeks apart [57]. Therefore, combination MDA programs could be a safe, clinically successful, logistically sound and cost-effective method for tackling multiple endemic disease in one community.

### Going forward

The identification of drivers of success of MDA programs for scabies may allow for the development of increasingly successful programs through evidence-driven MDA recommendations. For example, through increased oral Ivermectin use, ensuring high uptake (> 85%) is maintained, treating those contraindicated for oral therapy with topical regimens and following up those who are diagnosed with scabies at baseline. Moreover, recent evidence is demonstrating Ivermectin is safe for those weighing < 15 kg, so this could further revolutionize Ivermectin based MDA programs{Morris-Jones, 2020 #1684}. Similarly, MDA with moxidectin, an emerging scabies drug with a longer half-life, could further improve and simplify the success of MDA regimens [58]. The drivers of success suggested from this review could be further strengthened via modelling which could add valuable insights into further drivers of success and cost-effectiveness [59].

Another important aspect is ensuring the sustainability of MDA programs for scabies. Amongst the included studies the longest follow up period was 24 months. A 2 year follow up of one of the included studies in Fiji showed that at 24 months scabies prevalence remained reduced by 89% and this was similar in a 3 year follow up study in the Solomon islands [60, 61]. One study followed up, 15 years on, the effects of Ivermectin MDA for scabies and found that the initial baseline prevalence of 25% had remained reduced at 0.26% after 15 years [62]. However, the authors of these studies acknowledged that other interventions such as the reduction of overcrowding, increased education and access to scabies treatment had also had an impact on the reduction of scabies prevalence.

Therefore, pharmaceutical MDA regimens should be combined with sustainable interventions, such as, community education programs and the implementation of trained health professionals from which residents can seek treatment if they become infected. One community-based scabies program showed sustainable and effective reductions in scabies prevalence amongst a rural aboriginal community by implementing an educational program and environmental clean-up movement [63]. It is important to note that the control of scabies requires long term actions and that MDA is not to be used as a substitute to tackle the underlying issues that allow for scabies to become endemic such as overcrowding and poor hygiene. MDA should be part of a long-term plan that bridges this effective and quick strategy with long term eradication solutions like education, increased sanitation and increased healthcare access leading to empowerment within communities.

### Strengths and limitations

A strength of this review is the high quality of the tentwelve included studies. Seven (70%) of the included studies met over 75% of the NHLB checklist criteria and were categorised as high-quality studies. The studies generally had clear objectives, target population and MDA regimen. The main weaknesses identified using the NHLB checklist, such as lack of blinding, were not particularly relevant to the included studies which involved the whole population receiving the same treatment. Moreover, the majority of the studies were neither randomised nor controlled and we acknowledge this. Lastly, 50% of studies did not include a conflicts of interest statement, which could mean the non-disclosure of relationships that may influence judgements.

A limitation of the included studies is the geographical homogeneity of the included data which impacts its generalizability to other areas of the globe. Ten of the 12 included studies were based in rural communities in Oceania with the largest having only 5000 inhabitants . Moreover, the lack of evidence surrounding MDA programs for scabies in urban areas impact its generalizability. Urban areas endemic with scabies present barriers to MDA success such as increased migration, increased fear of adverse effects, and completion of secondary education, which all showed to be associated with decreased compliance [64].

Another limitation of the studies is the lack of long-term follow up. This limits our knowledge on the long-term effectiveness of MDA for scabies and the optimal time interval between repeated MDA administration to maximise scabies control. Other limitations of this review are those due to the inclusion and exclusion criteria. For example, the exclusion of unpublished literature and the exclusion of articles published before January 1990. However, these criteria were implemented to ensure a higher quality and relevance to the included data supported by peer-review and recency.

## Conclusion

In conclusion, this review highlights that MDA for scabies in endemic areas can be very successful in reducing the prevalence of scabies within communities. Some of the drivers of success for these interventions include the administration of oral medication, a high uptake in the community and lower levels of migration. Additionally, MDA programs targeting scabies can be beneficial to simultaneously combat other neglected tropical disease like lymphatic filariasis and intestinal helminths. Further evidence is required to analyse the success of MDA programs in diverse communities, for example urban areas with higher levels of migration, and to analyse the ideal frequency of administration to maintain low levels of disease.

It is crucial to note that MDA programs can provide a relatively quick and efficient method to substantially reduce the morbidity caused by scabies and its physical, mental and economic consequences within a community. Nevertheless, little evidence surrounding the cost-effectiveness of MDA programs for scabies is available. Moreover, MDA programs should not neglect the importance of non-pharmaceutical efforts for scabies control through the education, good sanitation and empowerment of local communities.

## Supplementary Information

**Additional file 1: Appendix S1**: Keywords used for Literature search. **Appendix S2**: Modified Tool ROBINS-I.

## Data Availability

The datasets used and/or analysed during the current study are available from the corresponding author on reasonable request.

## Abbreviations

S.Scabiei: Sarcoptes scabiei
GBD: Global Burden of Disease
DALY: Disability Adjusted Life Year
MDA: Mass drug administration
WHO: World Health Organisation
NHLB: National Heart, Lung & Blood
PRISMA: Preferred reporting items for a systematic review and meta-analysis protocol
