# Supplementary material for: Mass drug administration for endemic scabies: a systematic review
Source: Trop Dis Travel Med Vaccines. 2021 Jul 1;7:21. doi: 10.1186/s40794-021-00143-5 (PMC8247067; doi:10.1186/s40794-021-00143-5)
Supplement: Supplementary file 1 — Additional file 1: Appendix S1: Keywords used for Literature search. Appendix S2: Modified Tool ROBINS-I. [file 40794_2021_143_MOESM1_ESM.docx]

# Appendices

## Appendix S1: Keywords used for Literature search

| **Search strategy key words** |
| --- |
| ((((((((mass drug administration [Title]) OR administration [Title]) OR population[Title]) OR program*[Title]) OR community[Title]) OR regional[Title]))  AND ((scabies [Title]) OR infestation[Title]))  AND ((((((((effect*[Title]) OR reduc*[Title]) OR outcome [Title]) OR efficacy[Title]) OR treatment[Title]) OR prevalence[Title]) OR control[Title]) OR impact[Title]) |

## Appendix S2: Modified Tool ROBINS-I

| **Bias due to confounding** | |
| --- | --- |
|  | 1.1 Is there potential for confounding of the effect of intervention in this study?  **If N/PN to 1.1:** the study can be considered to be at low risk of bias due to confounding and no further signalling questions need be considered |
|  | **If Y/PY to 1.1**: determine whether there is a need to assess time-varying confounding: |
|  | 1.2. Was the analysis based on splitting participants’ follow up time according to intervention received?  **If N/PN**, answer questions relating to baseline confounding (1.4 to 1.6)  **If Y/PY**, go to question 1.3. |
|  | 1.3. Were intervention discontinuations or switches likely to be related to factors that are prognostic for the outcome?  **If N/PN**, answer questions relating to baseline confounding (1.4 to 1.6)  **If Y/PY**, answer questions relating to both baseline and time-varying confounding (1.7 and 1.8) |

|  | **Questions relating to baseline confounding only** |
| --- | --- |
|  | 1.4. Did the authors use an appropriate analysis method that controlled for all the important confounding domains? |
|  | 1.5. **If Y/PY to 1.4**: Were confounding domains that were controlled for measured validly and reliably by the variables available in this study? |
|  | 1.6. Did the authors control for any post-intervention variables that could have been affected by the intervention? |
|  | **Questions relating to baseline and time-varying confounding** |
|  | 1.7. Did the authors use an appropriate analysis method that controlled for all the important confounding domains and for time-varying confounding? |
|  | 1.8. **If Y/PY to 1.7**: Were confounding domains that were controlled for measured validly and reliably by the variables available in this study? |
|  | **Risk of bias judgement** |
|  | Optional: What is the predicted direction of bias due to confounding? |

| **Bias in classification of interventions** | |
| --- | --- |
|  | 2.1 Were intervention groups clearly defined? |
|  | 2.2 Was the information used to define intervention groups recorded at the start of the intervention? |
|  | 2.3 Could classification of intervention status have been affected by knowledge of the outcome or risk of the outcome? |
|  | **Risk of bias judgement** |
|  | Optional: What is the predicted direction of bias due to classification of interventions? |

| **Bias due to deviations from intended interventions** | |
| --- | --- |
|  | **If your aim for this study is to assess the effect of assignment to intervention, answer questions 3.1 and 3.2** |
|  | 3.1. Were there deviations from the intended intervention beyond what would be expected in usual practice? |
|  | 3.2. **If Y/PY to 3.1**: Were these deviations from intended intervention unbalanced between groups *and* likely to have affected the outcome? |
|  | **If your aim for this study is to assess the effect of starting and adhering to intervention, answer questions 3.3 to 3.6** |
|  | 3.3. Were important co-interventions balanced across intervention groups? |
|  | 3.4. Was the intervention implemented successfully for most participants? |
|  | 3.5. Did study participants adhere to the assigned intervention regimen? |
|  | 3.6. **If N/PN to 3.3, 3.4 or 3.5**: Was an appropriate analysis used to estimate the effect of starting and adhering to the intervention? |
|  | **Risk of bias judgement** |
|  | Optional: What is the predicted direction of bias due to deviations from the intended interventions? |

| **Bias due to missing data** | |
| --- | --- |
|  | 4.1 Were outcome data available for all, or nearly all, participants? |
|  | 4.2 Were participants excluded due to missing data on intervention status? |
|  | 4.3 Were participants excluded due to missing data on other variables needed for the analysis? |
|  | 4.4 **If PN/N to 4.1, or Y/PY to 4.2 or 4.3**: Are the proportion of participants and reasons for missing data similar across interventions? |
|  | 4.5 **If PN/N to 4.1, or Y/PY to 4.2 or 4.3**: Is there evidence that results were robust to the presence of missing data? |
|  | **Risk of bias judgement** |
|  | Optional: What is the predicted direction of bias due to missing data? |

| **Bias in measurement of outcomes** | |
| --- | --- |
|  | 5.1 Could the outcome measure have been influenced by knowledge of the intervention received? |
|  | 5.2 Were outcome assessors aware of the intervention received by study participants? |
|  | 5.3 Were the methods of outcome assessment comparable across intervention groups? |
|  | 5.4 Were any systematic errors in measurement of the outcome related to intervention received? |
|  | **Risk of bias judgement** |
|  | Optional: What is the predicted direction of bias due to measurement of outcomes? |

| **Bias in selection of the reported result** | |
| --- | --- |
|  | Is the reported effect estimate likely to be selected, on the basis of the results, from... |
|  | 6.1. ... multiple outcome *measurements* within the outcome domain? |
|  | 6.2 ... multiple *analyses* of the intervention-outcome relationship? |
|  | 6.3 ... different *subgroups*? |
|  | **Risk of bias judgement** |
|  | Optional: What is the predicted direction of bias due to selection of the reported result? |

| **Overall bias** | |
| --- | --- |
|  | **Risk of bias judgement** |
|  | Optional: What is the overall predicted direction of bias for this outcome? |

Original ROBINS-I tool available at: <https://methods.cochrane.org/bias/risk-bias-non-randomized-studies-interventions>
